# Motor Nerve Conduction Tests in Carpal Tunnel Syndrome

**DOI:** 10.3389/fneur.2019.00149

**Published:** 2019-03-14

**Authors:** Kristel M. Kasius, Franka Claes, Wim I. M. Verhagen, Jan Meulstee

**Affiliations:** ^1^Department of Neurology and Clinical Neurophysiology, Canisius Wilhelmina Hospital, Nijmegen, Netherlands; ^2^Department of Neurology and Clinical Neurophysiology, OLVG West, Amsterdam, Netherlands; ^3^Department of Neurology, Franciscus Vlietland Hospital, Schiedam, Netherlands

**Keywords:** carpal tunnel syndrome, nerve conduction studies, diagnostics, sensory nerve action potentials, motor nerve conduction tests

## Abstract

**Background:** For the preoperatively often required confirmation of clinically defined carpal tunnel syndrome (CTS), sensory as well as motor nerve conduction studies can be applied. The aim of this study was to test the sensitivity of specific motor nerve conduction tests in comparison with, as well as in addition to, sensory nerve conduction tests.

**Methods:** In 162 patients with clinically defined CTS, sensory and motor nerve conduction tests were performed prospectively. Sensitivity and specificity of all tests were computed. Also, Receiver Operating Characteristic (ROC) analyses were conducted.

**Results:** Sensitivity for all sensory tests was at least 79.4% (DIG1). All tests had a specificity of at least 95.7%. The motor conduction test with the highest sensitivity was the TLI-APB (81.3%); its specificity was 97.9%.

**Conclusion:** In the electrophysiological confirmation of CTS, sensory nerve conduction tests and terminal latency index have a high sensitivity. If, however, sensory nerve action potentials cannot be recorded, all motor nerve conduction tests have a high sensitivity.

## Introduction

Carpal tunnel syndrome (CTS) is the most common entrapment neuropathy (1, 2). Usually, the diagnosis can be reliably made on the basis of clinical signs and symptoms. In the Netherlands it is common that (neuro) surgeons require the clinical diagnosis to be confirmed electrodiagnostically prior to surgical treatment, which makes reliable electrodiagnostic tests an important issue (3).

It has previously been established that sensory nerve conduction studies are the most sensitive electrodiagnostic tests to confirm the diagnosis of CTS. Motor nerve conduction studies are important in the documentation of motor fiber involvement in CTS. In more severe cases, sensory nerve action potentials (SNAP) may not be recordable (4). In this case, motor nerve conduction studies are the only electrophysiological means to confirm the clinically defined diagnosis of CTS (5). However, median motor nerve conduction studies are supposed to be less sensitive than sensory nerve conduction studies (5). Several motor nerve conduction studies are available to confirm the diagnosis of CTS. The most commonly used test is the median distal motor latency (DML), obtained by recording over the abductor pollicis brevis (APB) muscle with stimulation at the wrist (1). Other motor nerve conduction studies are (1) the absolute value of the DML of the compound muscle action potential (CMAP) of the second lumbrical muscle (2) comparison of the motor latency of the CMAP of the lumbrical muscle with that of the interosseous muscle after stimulation of the median and ulnar nerve (6) and (3) the terminal latency index of the thenar CMAP (7). It has not been fully determined which motor nerve conduction study is the most reliable in the electrodiagnostic confirmation of CTS in patients whose sensory nerve action potentials cannot be recorded.

Therefore, in the present study we prospectively tested the sensitivity of both sensory as well as motor nerve conduction tests in a group of patients with clinically defined CTS. We particularly focused on the group of CTS patients whose sensory nerve action potentials could not be recorded; we tried to evaluate which motor nerve conduction test was the best alternative in these specific cases in terms of sensitivity.

## Materials and Methods

### Subjects

One hundred and sixty two patients with clinically defined CTS were included between 2006 and 2009. Data were collected prospectively. Carpal tunnel syndrome was considered to be clinically present in case of pain and/or paresthesias in the sensory distribution of the median nerve.

Two or more of the following criteria also needed to be present: (1) nocturnal paresthesias; (2) reproduction or aggravation of paresthesias or pain by provocative tests (Tinel or Phalen signs); aggravation of paresthesias by activities such as driving, riding a bike, holding a book or telephone; or (3) relief of symptoms by shaking the hand. These clinical criteria have previously been used in other studies (4, 8, 9). CTS with mild atrophy in combination with an MRC score ≥4 of the abductor pollicis brevis muscle and the opponens pollicis muscle was allowed.

In case of clinical signs of polyneuropathy or known hereditary neuropathy with a liability to pressure palsy, a history of trauma or any previous surgery to the symptomatic wrist, pregnancy, severe atrophy of the abductor pollicis brevis muscle, a history of rheumatoid arthritis or arthrosis of the wrist, known diabetes, thyroid disease, or alcoholism, patients were excluded from this study. Only the most symptomatic hand was included.

All candidates gave their written informed consent, and the local medical ethics committee approved the study (CWZ 1062006).

### Control Subjects

Reference values were derived from 47 healthy, asymptomatic volunteers, who were recruited from the hospital staff. All were tested in the same laboratory according to the same electrodiagnostic test protocol.

### Clinical Examination

All subjects underwent neurological examination, including inspection of the thenar, motor function tests of the hand muscles, the abductor pollicis brevis, and opponens pollicis muscle in particular, in accordance with Medical Research Council (10). Sensory tests, including a monofilament (10 g) and two-point discrimination were also performed. These data are not the subject of the present study.

### Electrodiagnostic Evaluation

All patients and healthy volunteers underwent standardized motor and sensory nerve conduction studies (NCS) in accordance with our laboratory's standard procedure as recommended by the American Association of Neuromuscular and Electrodiagnostic Medicine (AANEM) guidelines (1). Additionally, the residual motor latency was calculated (7). NCS were performed using a Viking Monograph IV (Nicolet Biomedical Inc. Madison, WI, USA). Skin temperature of the hands was maintained at a minimum of 31.0°C by means of hot packs (11) and it was measured before and after each test. One examiner, who was not informed of the preceding history and physical examination results, performed all tests.

#### Sensory Nerve Conduction Studies

Ring electrodes were applied for recording SNAPs. In all subjects, the proximal electrode was placed at the first interphalangeal joint and the distal electrode at a distance of, preferably, 3 cm. The optimal stimulation site was determined carefully. Signal averaging was applied on all SNAPs in order to obtain a sharp potential take-off from baseline or to ensure the SNAP was not recordable.

Conduction distances were measured with a precision of 1 mm, using a tape measure.

Three different antidromic sensory nerve conduction studies were performed: 2 comparison tests, 1 short segment study.

- DIG1: sensory median-radial comparison test: the median and radial nerves were stimulated separately at the wrist, and the SNAPs were recorded from the thumb. Onset latency differences were computed. SNAP amplitudes were measured (peak-to-peak).
- DIG4: sensory median-ulnar comparison test: the median and ulnar nerves were stimulated separately at the wrist, and SNAPs were recorded from the ring finger. Onset latency differences were computed. SNAP amplitudes were measured (peak-to-peak).
- PALM3: sensory short segment forearm-wrist vs. wrist-to-palm segment (4). SNAPs were recorded from the third finger after stimulation of the median nerve at the palm, wrist, and elbow, respectively. Differences in sensory nerve conduction velocities between the wrist-to-palm segments and forearm-to-wrist segments (forearm) were calculated using onset latencies.

#### Motor Nerve Conduction Studies

Compound muscle action potentials (CMAP) were recorded by means of surface electrodes. The recording position was chosen in a way that enabled recording a maximal CMAP, if possible, with a sharp initial negative deflection.

2 motor NCS were performed:

- DML-APB: distal motor latency (DML) to the abductor pollicis brevis muscle. The median nerve was stimulated at the wrist and at the elbow. CMAPs were recorded from the thenar eminence at a distance of 6 cm from the stimulation site. The reference electrode was positioned over the metacarpal-phalangeal joint of the thumb.
- 2L-INT: lumbrical-interosseous comparison study. The median and ulnar nerves were both stimulated at the wrist, with the same conduction distance. However, the value of the conduction distance varied per patient as the optimal stimulation site of the stimulus cathode was variable in order to be able to search for the optimal stimulation site (between 6 and 7 cm). CMAPs were recorded from the second lumbrical (2L) and second interosseous muscle (INT), respectively, with the active recording electrode in the palm, between the second and third metacarpals. The reference electrode was placed at the distal phalanx of the index finger. Distal motor latencies of the lumbrical (DML-LUMB) and interosseous (DML-INT) muscles were recorded, and differences between the two latencies were computed. The optimal recording site was defined as the location at which the CMAP was maximal with a sharp initial negative deflection.

For both DML-APB as well as 2L-INT terminal latency indexes (TLI) and residual motor latency (RML) were calculated by means of the following equations:

- TLI-APB = terminal distance (mm)/[motor conduction velocity forearm(m/s) ^*^ DML-APB (ms)]
- TLI-LUMB = terminal distance (mm)/[motor conduction velocity forearm(m/s) ^*^ DML-LUMB (ms)]
- RML-APB = DML-APB (ms) – [terminal distance (mm)/motor nerve conduction velocity forearm (m/s)]
- RML-LUMB = DML-LUMB (ms) – [terminal distance (mm)/motor nerve conduction velocity forearm (m/s)]

### Statistical Analysis

Data concerning clinical variables and nerve conduction studies were processed using Microsoft Office Excel and Access 2010; and all statistical analyses were performed using IBM SPSS Statistics 21.0.

In the reference group, mean differences, standard deviations, as well as upper and lower limits of normal (ULN and LLN, respectively) were calculated for all nerve conduction studies. ULN and LLN were defined as the mean, plus or minus twice the standard deviation, respectively.

The number of patients with an abnormal test result was determined using the ULN (or LLN in case of TLI). The sensitivity of each test was calculated as the number of patients that both met the criteria of clinical CTS *and* had an abnormal electrodiagnostic test result, divided by the number of patients meeting the criteria of clinical CTS times 100%. Specificity was calculated as the number of controls having normal test results, divided by the number of controls times 100%. Receiver Operating Characteristic (ROC) analyses were conducted. The Area Under the Curve (AUC) was computed for all nerve conduction studies.

Comparison between patients and the reference group was performed with a *t-*test for continuous variables or a χ^2^-test for categorical variables, as appropriate.

*P* < 0.05 values were considered as statistically significant test results.

## Results

### Clinical Features

One hundred and sixty two patients with clinical symptoms of CTS were included in this study, 35 men and 127 women. The mean age in this group was 48.7 (SD 13.6). The median duration of symptoms was 12 months. The mean age and gender distribution were significantly different between patients and controls (*P* < 0.01 and *P* < 0.05, respectively) (Table 1).

**Table 1:** Clinical features in patients and reference group.

	**Patients *n* = 162**	**Reference group *n* = 47**
Women	127 (78.4%)^*^	30 (63.8%)^*^
Age (mean ± SD, years)	48.73 ± 13.6^†^	41.04 ± 12.2^†^
Range (years)	18–86	19–59
Median symptom duration (months)	12.00	NA
Wrist included left/right	73 (45.1%)/89 (54.9%)	24 (51.1%)/23 (48.9%)^‡^
Sensory loss	125 (77.2%)	–
Monofilament	66 (40.7%)	–
Two-point discrimination	105 (64.8%)	–
Weakness abductor pollicis brevis muscle	47 (29.0%)	–
Weakness opponens pollicis muscle	10 (6.2%)	–

* *p ≤ 0.05*.

† *p ≤ 0.01*.

‡ *n = 87 (53.7%) bilateral complaints*.

*Sensory loss is defined as numbness reported by the patient at neurological examination by means of two-point discrimination and/or monofilament*.

### Electrophysiology

Details on electrophysiological features in both the patients and the reference group are presented in Table 2; ULN and LLN of performed tests are presented in Table 2.

**Table 2:** Electrophysiological features, sensitivity, and specificity.

	**Reference group (*****n*** **= 47)**		**Patients (*****n*** **= 162)**			
	**Mean ± SD**	**ULN/LLN^**†**^**	**Mean ± SD**	**Sens (%)**	**Spec (%)**	**AUC**
DIG1 (ms)	0.16 ± 0.19^*^	0.54	1.12 ± 0.73^*^	79.4	97.9	0.899
DIG4 (ms)	0.06 ± 0.14^*^	0.34	1.24 ± 0.95^*^	85.2	100	0.943
PALM3 (m/s)	5.64 ± 5.96^*^	17.6	25.3 ± 10.8^*^	81.8	100	0.931
DML-APB (ms)	3.36 ± 0.32^*^	4.0	5.23 ± 1.86^*^	71.0	100	0.900
2L-INT (ms)	0.08 ± 0.53^*^	1.13	1.86 ± 1.79^*^	58.2	95.7	0.874
TLI-APB	0.32 ± 0.03^*^	0.25	0.21 ± 0.06^*^	81.3	97.9	0.950
TLI-LUMB	0.39 ± 0.04^*^	0.30	0.28 ± 0.09^*^	69.5	95.7	0.858
RML-APB	2.27 ± 0.30^*^	2.86	4.11 ± 1.80^*^	73.8	100	0.911
RML-LUMB	1.99 ± 0.39^*^	2.77	3.63 ± 1.67^*^	66.9	95.7	0.860

*Numbers may vary due to missing values or not recordable SNAPs or CMAPs*.

* *p ≤ 0.01*.

† *LLN applies to TLI-APB and TLI-LUMB only*.

*ULN, upper limit of normal; LLN, lower limit of normal; AUC, area under the curve; DIG1, sensory median-radial comparison test; DIG4, sensory median-ulnar comparison test; PALM3, sensory short segment forearm-wrist vs. wrist-to-palm segment; DML-APB, distal motor latency to the abductor pollicis brevis muscle; 2L-INT, lumbrical-interosseous comparison study; TLI-APB, terminal latency index abductor pollicis brevis muscle; TLI-LUMB, terminal latency index lumbrical muscle; RML-APB, residual motor latency abductor pollicis brevis muscle; RML-LUMB, residual motor latency lumbrical muscle*.

The DML to the APB was 3.36 ± 0.32 and 5.23 ± 1.86 ms (mean ± SD) in the reference group and patients, respectively. 2L-INT was 0.08 ± 0.53 ms in the reference group vs. 1.86 ± 1.79 ms in patients. DML-APB was abnormal in 115 patients (70.6%); in 5 of them the CMAP was not recordable. 2L-INT was abnormal in 92 of 158 (58.2%), and in 4 of these the lumbrical CMAP was not recordable. These differences were statistically significant (*P* < 0.01).

TLI-APB was abnormal in 130 of 160 patients (81.3%), TLI-LUMB in 107 of 154 (69.5%). RML-APB was abnormal in 118 of 160 patients (73.8%), RML-LUMB in 103 of 154 (66.9%) (Table 2, *P* < 0.01). The number of tests varies between APB and LUMB because of missing values.

**Figure 1:**
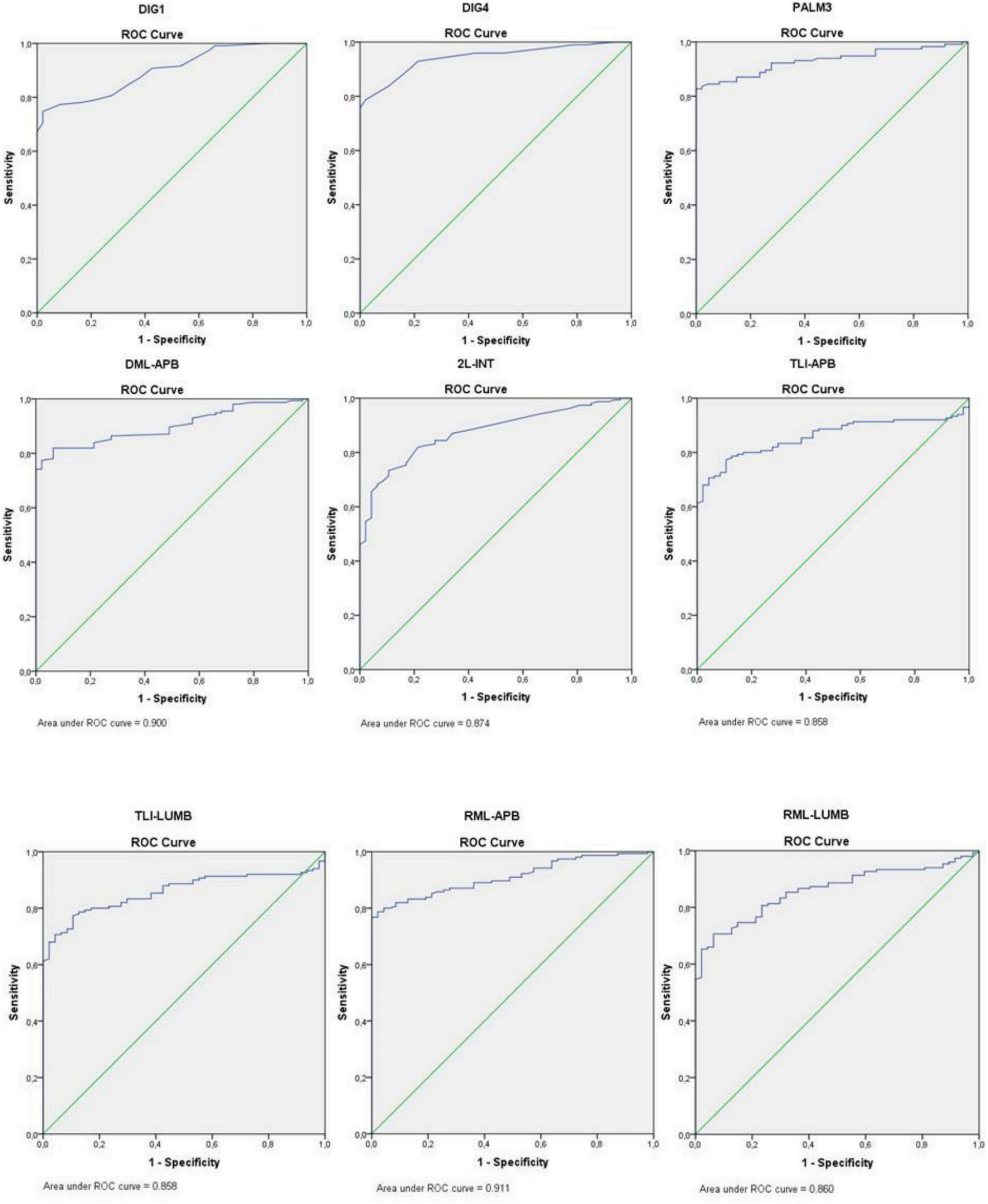
ROC curve nerve conduction studies. DIG1, sensory median-radial comparison test; DIG4, sensory median-ulnar comparison test; PALM3, sensory short segment forearm-wrist vs. wrist-to-palm segment; DML-APB, distal motor latency to the abductor pollicis brevis muscle; 2L-INT, lumbrical-interosseous comparison study; TLI-APB, terminal latency index abductor pollicis brevis muscle; TLI-LUMB, terminal latency index lumbrical muscle; RML-APB, residual motor latency abductor pollicis brevis muscle; RML-LUMB, residual motor latency lumbrical muscle.

Sensitivity for all sensory tests was at least 79.4% (DIG1); for motor conduction tests sensitivity was considerably lower, except for the TLI-APB, which was 81.3%. All conduction tests had high specificity values (range 95.7–100%) with AUC values ranging from 0.858 to 0.950. Of the motor nerve conduction tests, it was the TLI-APB that had the highest sensitivity (81.3%), a high specificity (97.9%), and a high AUC value (0.950) (Figure 1).

In CTS patients with no recordable SNAPs [meaning that no median nerve SNAP could be recorded from DIG1, DIG4, and PALM3 (*n* = 27) with stimulation at the wrist], the percentage of abnormal motor nerve conduction tests was 100. This was significantly more than in patients with recordable SNAPs (*P* < 0.01).

Numbers of normal and abnormal tests according to 2L-INT and DML-APB are presented in Table 3. Out of 47 CTS patients with normal DML-APB test results, only 2 patients had an abnormal 2L-INT test result. In contrast, 23 of 68 patients with normal 2L-INT test results had an abnormal DML-APB test result (Table 3).

**Table 3:** Lumbrical-interosseous (2L-INT) vs. DML-APB in all patients.

		**2L-INT**							
		**Normal**		**Abnormal**		**CMAP not recordable**		**Total**	
DML-APB	Normal	45		2		0		47	(29.4%)
	Abnormal	23		85		0		108	(67.5%)
	CMAP not recordable	0		1		4		5	(3.1%)
	Total	68	(42.5%)	88	(55.0%)	4	(2.5%)	160	(100%)

*2L-INT, lumbrical-interosseous comparison study; DML-APB, distal motor latency to abductor pollicis brevis muscle; CMAP, compound muscle action potential*.

## Discussion

Our results confirm that out of all electrodiagnostic tests that are available to confirm the clinical diagnosis of CTS, sensory conduction studies are the most sensitive (1, 5). In severe CTS, however, sensory nerve action potentials often cannot be recorded. Motor nerve conduction studies have to be performed to show the presence of conduction slowing in the median nerve across the wrist, in order to differentiate between CTS and for example a proximal lesion of the median nerve. Several types of motor conduction studies can then be applied to show the presence of conduction slowing in the median nerve across the wrist. Most often, the distal motor latency of the median nerve is used, defined as the onset latency of the APB CMAP, which is obtained by stimulation of the median nerve at the crease of the wrist. A cut-off value of 4.0 ms without taking conduction distance into account, has a reported sensitivity of 6 to 65% (1).

In theory, a better alternative is the lumbrical-interosseous comparison test as described in the methods. The comparative aspect of this test is advantageous, as the motor conduction of the ulnar nerve is its reference. One may expect that nerve conduction velocities in distal segments of median and ulnar nerves do not differ much (12). In our data, however, the sensitivity of this test in the whole group of patients is even less than that of the classic DML-APB test. Moreover, 23 of 68 patients with a normal lumbrical-interosseous comparison test result showed an abnormal DML-APB. In the subgroup of patients with more severe CTS, whose SNAPs are not recordable, we found that all specific motor nerve conduction tests show a high sensitivity of up to 100%. In only one patient the CMAP to the APB was not recordable while the lumbrical-interosseous comparison test showed abnormal results, which proved a distal median neuropathy. The sensitivity which was found in this study is considerably lower compared to reported sensitivities in the literature (12, 13). This difference can be explained by the different cut-off values. Preston et al. (6) used a cut-off of 0.4 ms, and Chang et al. (13) used 0.6 ms. According to our reference population the cut-off value is 1.13 ms. We do not have a satisfying explanation for this difference.

The great advantage of the lumbrical-interosseous comparison test over the other motor nerve conduction studies is, that a DML of ulnar muscles can be used as a reference for the thenar DML. However, since in only one patient the lumbrical-interosseous comparison test had additional value to the DML-APB, we could not confirm the hypothesis that motor fibers to the lumbrical muscle at the level of the carpal tunnel are less vulnerable because of their anatomical/topographical position in the median nerve, which has been suggested by others (6, 12–17). Moreover, the association between 2L-INT and DML-APB is high (*r* = 0.87, *P* < 0.001), making the additional value of the 2L-INT to the traditionally performed DML only marginal. Values in the same order of magnitude were found in the subgroup of CTS patients whose SNAP could not be recorded.

As TLI gives a DML correction to nerve conduction velocity in the proximal segment of the median nerve as well as in the terminal distance, it is not very surprising that we found that the TLI of APB showed a high sensitivity, almost similar to sensory tests. This goes for the whole patient group as well as the subgroup of patients whose SNAPs are not recordable. This is in accordance with previous reports (7, 17–19), yet normative values may vary because of methodological differences and the electrophysiological techniques used. One may argue that, considering the tortuous course of the median nerve in the carpal tunnel (20) it is virtually impossible to measure the distal conduction distance precisely. As a consequence, the TLI value may be biased to lower values as it is to be expected that measured distances are underestimated. However, this is not an issue, since the same argument can be used for the TLI value acquired in healthy subjects, if reference values are collected in the same way as in the patients, which is the case in our study.

The conclusions of our study apply to patients with no or mild thenar atrophy. We excluded patients with severe thenar atrophy, which is probably the group of patients with the most severe CTS. Numerically, this is presumably not a very significant group of patients; according to the study of Yates et al. (16), only 5% of the total carpal tunnel syndrome population tested in the laboratory over a 2 and a half year period had severe thenar wasting. It could therefore be worthwhile to investigate the group of very severe CTS patients with the aforementioned tests.

Since we used the clinical diagnosis as the standard, and all patients in this study had clinical CTS, specificity could not be calculated. Knowledge of specificities of motor nerve conduction studies would have been of additional value. Also, the reference group was not completely matched for age and sex with the patient group and this may have influenced the results. However, when comparative tests within the same subject are used, differences in age or sex are probably less relevant.

In conclusion, it appears that in CTS patients with recordable SNAPs, motor nerve conduction tests are less sensitive to confirm the clinical diagnosis of CTS with the exception of the TLI of the APB. In CTS patients whose SNAPs are not recordable, all discussed motor tests have a high sensitivity. The TLI in particular, appears to be a robust electrodiagnostic test in CTS.

In cases when median nerve SNAPs cannot be recorded, but a CMAP to the APB can, the 2L-INT has no additional value. However, in our study the chance of recording a lumbrical CMAP in these specific cases is rather low.

## Data Availability

The datasets for this manuscript are not publicly aware because several other papers related to the database are pending.

## Author Contributions

KK: data processing and writing the manuscript. FC, WV, and JM: study design, data collection, and review the manuscript.

### Conflict of Interest Statement

The authors declare that the research was conducted in the absence of any commercial or financial relationships that could be construed as a potential conflict of interest.
